# Amazonian Native Palm Fruits as Sources of Antioxidant Bioactive Compounds

**DOI:** 10.3390/antiox4030591

**Published:** 2015-09-07

**Authors:** Mary de Fátima Guedes dos Santos, Rosa Virginia Soares Mamede, Maria do Socorro Moura Rufino, Edy Sousa de Brito, Ricardo Elesbão Alves

**Affiliations:** 1 Institute of Scientific and Technological Research of the State of Amapá (IEPA), Rodovia JK, Km 10, Macapá, AP 68900-000, Brazil; E-Mail: mary_guedes_ap@hotmail.com; 2 Embrapa Tropical Agroindustry, R. Dra. Sara Mesquita, 2270, Pici, Fortaleza, CE 60511-110, Brazil; E-Mail: ricardo.alves@embrapa.br; 3 Deparment of Chemistry, Universidade Estadual do Piauí, Av. Presidente Castelo Branco, 180, Piripiri, PI 64260-000, Brazil; E-Mail: rosavsoares@yahoo.com.br; 4 Institute of Rural Development, University for the International Integration of the Afro-Brazilian Lusophony, Av. da Abolição, 3, Centro, Redenção, CE 62790-000, Brazil; E-Mail: marisrufino@unilab.edu.br

**Keywords:** *Oenocarpus bacaba*, *Mauritia flexuosa*, *Attalea maripa*, *Bactris gasipaes*, *Astrocaryum vulgare*

## Abstract

The Amazon region has many sources of fruits, especially native ones not yet explored, but which have some potential for use, as is the case with certain palms. The objective of this study was to evaluate the content of bioactive compounds and total antioxidant capacities of fruits from native palms from the Brazilian Amazon. The fruits of five palm species (*bacaba*, *buriti*, *inajá*, *pupunha*, and *tucumã*) were evaluated for levels of ascorbic acid, anthocyanins, yellow flavonoids, total carotenoids, and total extractable polyphenols, as well as the total antioxidant capacities. The fruits had high contents of extractable total polyphenols, especially *bacaba* and *tucumã* (941.56 and 158.98 mg of galic acid·100g^−1^), total carotenoids in the case of *tucumã* and *buriti* (7.24 and 4.67 mg·100g^−1^), and anthocyanins in *bacaba* (80.76 mg·100g^−1^). As for the antioxidant capacity, *bacaba* had the highest total antioxidant activity by the Oxygen Radical Antioxidant Capacity (ORAC) (194.67 µM·Trolox·g^−1^), 2,2-diphenyl-1-picrylhydrazyl (DPPH) (47.46 g·pulp·g^−1^ DPPH), and β-carotene/linoleic acid (92.17% Oxidation Inhibition (O.I) methods. *Bacaba* phenolic profile revealed the presence of cyanidin-3-*O*-rutinoside and other flavonoids. The palm fruits studied can be considered good sources of bioactive compounds, some containing higher amounts than that of commonly consumed fruits. Total extractable polyphenols and anthocyanins were directly correlated to antioxidant activity in these fruits.

## 1. Introduction

Fruits have chemical compounds that play important biological roles. These, called bioactive or phytochemical compounds, are usually found in small amounts in foods, especially plants, and may have different beneficial ways of acting on human health [1]. According to [2], fruits are known as functional foods; besides providing nutrition for the proper functioning of organisms, their value is associated with substances that have protective properties, in most cases due to their antioxidant activity. Among these substances, the polyphenols—carotenoids, ascorbic acid, phytosterols, *etc.—*stand out. Due to their antioxidant properties, these compounds slow down the oxidant reactions, thus protecting the organism against reactive oxygen species [3]. 

In recent years, the search for foods that are good sources of natural antioxidants has been driving studies to evaluate compounds present in plant species that could be related to antioxidant activity [4]. The Amazon region hosts numerous native fruits that might fill in this gap and have the potential to be explored as functional foods, among which are some Arecaceae species such as *bacaba* (*Oenocarpus bacaba*), *buriti* (*Mauritia flexuosa*), *inajá* (*Attalea maripa*), *pupunha* (*Bactris gasipaes*), and *tucumã* (*Astrocaryum vulgare*). Recently, those species have been studied for oil and carotenoids composition [5,6,7]. However, there is little scientific information on the functional potential of native palms, especially those that are underutilized as food sources in the region. Studies listing the functional compounds in these species, as well as their antioxidant capacity, may incentivize their consumption, besides generating new usage alternatives, which adds value to these plants. Therefore, the goal of this study was to assess the levels of bioactive compounds and the total antioxidant capacity of native Amazon rainforest palm fruits aiming to identify their functional components and thus value their consumption.

## 2. Experimental Section 

### 2.1. Chemicals

The reagents used were 2,6-dichlorophenolindophenol (DFI), ethyl alcohol, hydrochloridric acid, isopropyl alcohol, hexane, anhydrous sodium sulfate, acetone, methyl alcohol, Folin-Ciocalteu reagent (FCR), sodium carbonate, 6-hydroxy-2,5,7,8-tetramethylchroman-2-carboxylic acid (Trolox), ethyl ether, β-carotene, linoleic acid, chloroform, 2,2-diphenyl-1-picrylhydrazyl (DPPH), potassium phosphate monobasic (KH_2_PO_4_), sodium phosphate dibasic anhydrous (Na_2_·HPO_4_), AAPH radical, and fluorescein (3′,6′-dihydroxyspiro(isobenzofuran-1[3H], 9′-[9H]xanthen]-3-one). High Pressure Liquid Chromatography (HPLC) grade acetonitrile and methanol were purchased from Tedia Company Inc. (Fairfield, OH, USA). HPLC grade water was prepared from distilled water using a Milli-Q system (Millipore Lab., Bedford, MA, USA).

### 2.2. Sample Preparation and Chemical Analysis

Ripe fruits from native Amazonian palm trees (Table 1) were harvested, after the natural process of abscission, in different regions of Amapá State, Brazil, according to maturity stages described in Table 2. The fruits were harvested from one tree, selected and distributed in three batches containing 27 fruits each for *buriti*, *inajá*, *pupunha* and *tucumã* and 50 fruits for *bacaba*. Each batch was processed in three analytical replicates. In *bacaba*, the skin is inseparable from the pulp, so it was considered as edible. In *tucumã*, the edible portion includes both the mesocarp and the epicarp, which is the traditional way the fruit is consumed in the region. For *buriti*, *inajá*, and *pupunha*, only the mesocarp was analyzed. The samples were lyophilized and stored at –70 °C until analysis and moisture content was determined for all fruits. The results were expressed on a fresh weight basis.

**Table 1:** List of Arecaceae from Amazon region, Amapá State, Brazil, included in this study.

Local Name	English Name	Scientific Name	Origin (City)
Bacaba	Turu Palm	*Oenocarpus bacaba* Mart.	Porto Grande
Buriti	Moriche Palm	*Mauritia flexuosa* L.f.	Mazagão
Inajá	American oil Palm	*Attalea maripa* (Aubl.) Mart.	São Joaquim do Pacuí
Pupunha	Peach Palm	*Bactris gasipaes* Kunth.	Porto Grande
Tucumã	Awarra Palm	*Astrocaryum vulgare* Mart.	Macapá

**Table 2:** Harvest maturity stages of Amazon Arecaceae fruits.

Fruits	Harvest Maturity Stages
Bacaba	Dark purple peel and white-brown pulp
Buriti	Yellow-orange pulp
Inajá	Cream-yellowish pulp
Pupunha	Lightly orange pulp
Tucumã	Dark orange peel and pulp

Vitamin C content was determined through the 2,6-dichlorophenolindophenol method [8], total anthocyanins and yellow flavonoids as described by [9], and total carotenoids according to [10].

### 2.3. Sample Preparation and Total Polyphenols

Lyophilized samples stored at −70 °C were used to obtain the extracts. Preliminary tests were carried out in order to determine the ideal amount of samples in the extracts. The final dry masses used in the extracts from *buriti*, *inajá*, *pupunha*, and *tucumã* were 5 g, while for *bacaba* it was 2 g. Before extraction, the samples were grounded on a mortar with liquid nitrogen. The procedure used was developed by [11]. Briefly, the samples were weighed in test tubes and 20 mL of methanol/water (50:50, v/v) was added for the extraction. The tubes were left to rest at room temperature for 1 h and centrifuged at 15,000 rpm for 15 min, and after that the supernatant was collected. Subsequently, 20 mL of water/acetone (70:30, v/v) was added to the residue for extraction for 1 h at room temperature, followed by centrifugation. The methanol and acetone extracts were combined, completed up to 50 mL with distilled water, and used in determining total polyphenol content and antioxidant capacity.

In order to determine total polyphenols, the Folin-Ciocalteu method [12] was followed. To 1.0 mL of the extracts, 1 mL of Folin-Ciocalteu reagent (1:3), 2 mL of a 20% sodium carbonate solution, and 2 mL of distilled water were added. After 30 min, a reading was performed at 700 nm. The results were expressed as Gallic Acid Equivalents (GAE) (mg GAE 100 g^−1^).

2.4. β-Carotene/Linoleic Acid Method

The antioxidant capacity was evaluated according to the method developed by [13] with some adaptations. The samples were prepared using 5 mL of the β-carotene/linoleic acid system solution, 0.4 mL of Trolox control solution, and 0.4 mL of the fruit extracts at different dilutions. Readings in a spectrophotometer at 470 nm were performed immediately and in 15-minute intervals for 120 minutes with the tubes kept in a water bath at 40 °C. The results were expressed as the percentage of oxidation inhibition and the fruits antioxidant capacities were assessed by comparing them to the control solution (Trolox).

### 2.5. DPPH Method (Ability to Scavenge Free Radicals)

The fruit’s antioxidant capacity was determined through the method developed by [14], which is based on scavenging the DPPH radical (2,2-diphenyl-1-picrylhydrazyl) by antioxidants in the extracts, with some modifications. A DPPH solution in methanol at 0.06 mM was prepared. Then, 100 µL aliquots of the extracts were transferred to test tubes, 3.9 mL of the DPPH solution was added, and a reading at 515 nm was performed. The reduction in absorbance was monitored every minute in the first 10 min and every five min until the mixture fully stabilized. This way, the kinetics equivalent to the time needed for each sample to consume 50% of the free radical (EC_50_) was obtained. The results were 35 min for *bacaba*, 90 min for *buriti*, *inajá*, and *pupunha*, and 120 min for *tucumã*. The antioxidant activity was expressed by the antioxidant concentration required to reduce the initial free radical concentration by 50% and the final result was expressed in g of fresh weight of edible portion of the fruit·g^−1^ DPPH.

### 2.6. ORAC (Oxygen Radical Absorbance Capacity)

Antioxidant activity was determined according to the methodology by [15] with adaptations. The fruit extracts were prepared by mixing 0.5 g of the lyophilized sample and 20 mL of acetone/water (50:50, v/v), which were kept at room temperature for 1 h and then centrifuged at 25,000 g for 15 min. The supernatant was filtered and stored under freezing conditions until the time of analysis. For each species evaluated, preliminary tests were performed so as to obtain distinct concentrations using a phosphate buffer solution (75 mM, pH 7.4). A standard Trolox curve was prepared at five concentrations (6.25; 12.5; 25; 50, and 100 µM). The samples were prepared on the micro plate, 250 µL of the fluorescein solution (48 nM) were added and the micro plate were kept in the incubator at 37 °C for 10 min. Next, 50 µL of the AAPH radical (153 mM) were added to each sample. The readings were performed using the Kinetics program of the Varian Cary Eclipse Fluorescence Spectrophotometer and the reduction in fluorescein was monitored every minute for 180 min. The ORAC values were calculated from the area under the emission curve of fluorescein, which is proportional to the Trolox concentration. The final result was expressed as µM of Trolox equivalents by gram of the sample (μM·Trolox·g^−1^).

### 2.7. LC-DAD-ESI-MS Analysis

The general procedure for screening of phenolics in plant materials [16] was employed with modifications, except for acid hydrolysis. The LC-DAD-ESI/MS instrument consisted of a Varian 250 HPLC (Varian, Walnut Creek, CA, USA) coupled with a diode array detector (DAD) and a 500-MS IT mass spectrometer (Varian, Walnut Creek, CA, USA). A Zorbax C18 (Agilent, Little Falls, DE, USA) column (5 μm, 150 × 2 mm) was used at a flow rate of 0.4 mL/min. The column oven temperature was set to 30 °C. The mobile phase consisted of a combination of A (0.1% formic acid in water) and B (0.1% formic acid in acetonitrile). The gradient varied linearly from 10%–26% B (v/v) at 40 min, to 65% B at 70 min, and finally to 100% B at 71 min and remained at 100% B for 75 min. The DAD was set at 350, 270, and 520 nm for real-time read-out, while the UV/VIS spectra, from 190–650 nm, were continuously collected. Mass spectra were simultaneously acquired using electrospray ionization in the positive (PI) and negative ionization (NI) modes at a fragmentation voltage of 80 V for the mass range of 100–2000 amu. A drying gas pressure of 35 psi, nebulizer gas pressure of 40 psi, a drying gas temperature of 370 °C, capillary voltages of 3500 V for PI and 3500 V for NI, and spray shield voltages of 600 V were used.

### 2.8. Statistical Analysis

The trials were carried out in triplicate and the results underwent a descriptive statistical analysis for obtaining the average values and the standard deviation for each palm species studied. Pearson’s correlation analysis at 1% and 5% significance levels by t-test was also performed among the bioactive compounds (ascorbic acid, total anthocyanins, yellow flavonoids, total carotenoids, and total polyphenols) and the total antioxidant activity through the β-carotene/linoleic acid, DPPH, and ORAC methods using the GENES software (UFV, Viçosa, Brazil).

## 3. Results and Discussion

The results obtained for Vitamin C, total anthocyanins, yellow flavonoids, and total carotenoids are presented in Table 3 along with the percentage of moisture. Açaí, one of the most studied Amazon palm fruits, [17] reported a Vitamin C content of 80 mg·100g^−1^ of fresh weight, which is well above the results found in this study for any of the five palm species. Vitamin C is a crucial compound in many physiological processes in the body and, in plants, has a protective role against reactive oxygen types formed in the respiratory and photosynthesis processes. Therefore, even the fruits that have relatively low amounts of Vitamin C, such as *pupunha* and *buriti*, should be valued. 

**Table 3:** Bioactive compounds (mg·100g^−1^ of fresh weight) and moisture (%) in edible parts of five Amazon native fruits from palm trees.

Fruits	Vitamin C	Total Anthocyanins	Yellow Flavonoids	Total Carotenoids	Moisture
Bacaba	30 ± 2	81 ± 1	36 ± 2	0.7 ± 0.1	42 ± 2
Buriti	13 ± 1	3 ± 0.4	28 ± 2	4.7 ± 0.1	62 ± 3
Inajá	24 ± 2	1 ± 0.2	14 ± 0.2	0.4 ± 0.0	59 ± 5
Pupunha	14 ± 1	1 ± 0.1	17 ± 1	2.6 ± 0.2	65 ± 1
Tucumã	19 ± 1	4 ± 0.2	31 ± 2	7.2 ± 0.4	56 ± 2

Anthocyanins are flavonoids, compounds containing phenolic hydroxyls, and are described as potential antioxidants since they have an ideal structure for radical scavenging. *Bacaba* had a total anthocyanin content of 81 mg·100·g^−1^, comparable to that found by [17] in other fruits from the Arecaceae family (açai and juçara palm), which are well-known sources of anthocyanins with average contents of 111 and 93 mg·100·g^−1^, respectively. Abadio Finco *et al.* [18] characterized the phenolic extract of *bacaba* and reported a content of 1759 (mg·GAE/100·g) for total phenolics, 1134 (mg·CTE/100·g) for total flavonoids and 35 (mg cyn-3-glc/100g) for total anthocyanins, indicating that *bacaba* is a promising source of phenolic compounds. Schauss *et al.* [19], while studying the phytochemical composition of açai, reported that this fruit is rich in anthocyanins, with cyanidin 3-glucoside and cyanidin 3-rutinoside being the main ones. Thus, due to the high total anthocyanin content found in *bacaba*, this fruit may be considered a potential source of antioxidants.

Anthocyanins and flavonols are part of the flavonoid group and are responsible for the color ranging from bright red to violet, and from white to light yellow, respectively. Among the fruits studied, those that had the highest levels of these compounds (*bacaba*, *tucumã*, and *buriti*) were the ones that had the most intense color in the analyzed portion. Flavonoids act as antioxidants in inactivating free radicals in both the lipophilic and hydrophilic cell compartments [20]. Studies have shown that flavonoids from different sources have beneficial health effects, acting as anti-inflammatories, anti-atherosclerotics, and immunomodulators [21,22].

Among the fruits studied, *tucumã* and *buriti* stood out from the rest for having the highest total carotenoid contents at 7.2 and 4.7 mg·100 g^−1^, respectively. Mambrim and Barrera-Arellano [23], characterized fruits from Amazonian palms, among which *bacaba* and *tucumã*, reported total carotenoid values of 0.29 and 2.42 mg·100 g^−1^, respectively, which are well below those found in the present study. Rodriguez-Amaya [24] reported that *buriti* and African oil palm fruits stand out as the richest pro-Vitamin A sources found in Brazil. Therefore, beside *bacaba* and *inajá*, the fruits of the other species studied (*pupunha*, *buriti*, and mainly *tucumã*) may be considered sources of these compounds. 

Polyphenol contents greatly varied among the species studied (Table 4). *Bacaba* stood out for having higher polyphenol content (941 mg GAE·100 g^−1^ of fresh weight), suggesting these fruits are excellent sources of polyphenols and that many of these compounds are likely associated with the fruit’s color. In *tucumã* and *buriti*, average amounts of TEP were found (159 and 118 mg GAE·100 g^−1^). Meanwhile, *pupunha* and *inajá* had low polyphenol contents. In the study by Rufino *et al.* [17], which comprised 18 non-traditional Brazilian tropical fruits, including two Arecaceae (juçara and açai), high polyphenol contents were reported (755 and 454 mg GAE·100 g^−1^, respectively). These levels, however, are still below those found in *bacaba*.

**Table 4:** Total polyphenols and antioxidant capacity in organic-aqueous extracts of five Amazon native fruits from palm trees (fresh weight) ^a^.

Fruits	Total Polyphenols mg GAE·100 g^−1^	β-Carotene/Linoleic Acid % O.I. ^b^	DPPH EC_50_ (g·g^−1^DPPH) ^c^	ORAC ^d^ (μM Trolox·g^−1^)
Bacaba	941 ± 23	92 ± 1	47 ± 1	195 ± 10
Buriti	118 ± 2	65 ± 3	7938 ± 121	89 ± 6
Inajá	45 ± 2	80 ± 2	18,936 ± 252	26 ± 2
Pupunha	30 ± 1	62 ± 1	nd	94 ± 1
Tucumã	159 ± 13	92 ± 0	3343 ± 132	64 ± 4

^a^ Average values ± standard deviation, *n* = 3; nd = not determined. ^b^ Oxidation inhibition. ^c^ Antioxidant. ^d^ Oxygen Radical Antioxidant Capacity. Concentration required to reduce free radical concentration by 50%.

Roesler *et al.* [25], while studying fruits from the Brazilian Cerrado ecosystem, found that the fractions with the highest phenolic compound contents were the skins and seeds, which are usually discarded when the fruit is eaten fresh. In the current study, the edible portion analyzed of *bacaba* and *tucumã* included both the mesocarp and the epicarp, which may likely have influenced the high polyphenol contents found in these fruits compared with the other fruits studied. 

The results of antioxidant capacity determined by the β-carotene/linoleic acid, DPPH, and ORAC methods are shown in Table 3. In the present study, antioxidant activity was evaluated in terms of the ability of the compounds present in the fruit extracts to prevent oxidation of β-carotene, protecting it from free radicals generated during the peroxidation of linoleic acid. Based on the study by Hassimotto, Genovese & Lajolo [26], the antioxidant capacity in plants and fruit pulps can be classified, according to the levels of oxidation inhibition (O.I.), as high (>70%), intermediate (40%–70%), or low (<40%). Among the fruits studied, *bacaba*, *tucumã*, and *inajá* had high antioxidant capacity (92%, 92%, and 80% O.I., respectively), while *buriti* and *pupunha* had intermediate antioxidant capacity (65% and 62% O.I., respectively). It is worth pointing out that, although the concentration of the extract used was much lower for *bacaba*, the percentage of oxidation inhibition was high, which shows the strong antioxidant capacity of that fruit compared to the other palm species studied.

Hassimotto, Genovese & Lajolo [26], while studying the antioxidant capacity of different fruit pulps, among which açai, through the β-carotene/linoleic acid method, reported no significant correlation between antioxidant capacity and concentration of phenolics. On the other hand, Duarte Almeida *et al.* [27], while evaluating the antioxidant capacity in pure phenolic compounds and pulp extracts through the β-carotene/linoleic acid method, found that fruits such as açai, strawberry, and mulberry, which have the highest flavonoid contents, had high antioxidant capacity, being quercertin the one showing the highest oxidation inhibition percentage even in smaller amounts. For Koleva *et al.* [28], the exact mechanism of action of the antioxidant in the β-carotene/linoleic acid method was hard to explain, especially when testing the action of complex matrices such as plant extracts, including fruits. In trials using lipids as oxidizable substrate, as is the case of the β-carotene/linoleic acid method, the protective role of the antioxidant depends on its solubility, which determines its distribution in the system phase, including location and orientation [29]. Just as well, the complex composition of plant extracts may cause synergic or antagonistic interactions among the compounds, which may also affect their antioxidant activity [30].

When evaluating the antioxidant capacity through the DPPH method, the amount of antioxidant present in the extracts able to scavenge 50% of the free radicals in the solution (EC_50_) was determined. The lower this value, the lower the amount of extract required to reduce by 50% the DPPH free radical and, consequently, the higher its antioxidant capacity. Among the fruits studied, *bacaba* stood out for having the highest antioxidant capacity (47 g·g^−1^ DPPH), followed by *tucumã* (3343 g·g^−1^ DPPH). The antioxidant capacity shown by *bacaba*, *tucumã*, and *buriti* are higher than those reported by Rufino *et al.* [17] for two Areacaceae species: açai (4264 g·g^−1^ DPPH) and juçara (1711 g·g^−1^ DPPH). It is worth noting that, in the present study, the fruits that stood out for their antioxidant capacity were the same ones that had the highest polyphenol contents, suggesting a relation between the phenolic compounds and the antioxidant capacity. For *pupunha*, no satisfactory results were found that allowed the evaluation of the antioxidant capacity through the DPPH method despite countless trials at different concentrations. It was considered that the low polyphenol levels in this fruit may be one of the factors contributing to this result.

Through the ORAC method, *bacaba* showed the highest antioxidant capacity (195 µM·Trolox·g^−1^), while the lowest was found for *inajá* (26 µM·Trolox·g^−1^). All the results matched those found through the DPPH method. The high antioxidant capacity shown by *bacaba* may be related to the high total polyphenol contents, which are directly associated to the antioxidant capacity. Abadio Finco *et al.* [18] evaluated the antioxidant activity of *bacaba* through ORAC and DPPH methods and got values of 10,750 (µM·Trolox·100g^−1^) and 34 (mM Trolox·100g^−1^), respectively, which confirms the significant antioxidant capacity of this fruit, finding a value higher than the one in this study.

Schauss *et al.* [19], while studying the antioxidant capacity of açai through different assays and with several free radical sources, found a high ORAC value (997 µM·Trolox·g^−1^ of dry weight). That study stresses the high antioxidant capacity shown by this fruit. *Bacaba*, even having a lower value than açai, has a high antioxidant capacity when compared to most fruits. Except for *bacaba*, to our knowledge there are no references in the literature about antioxidant capacity determined by the ORAC method for the other species studied. However, Wu *et al.* [31], while evaluating total antioxidant capacity combining the lipophilic and hydrophilic fractions of the components in 100 different types of foods, including fruits, found that, except for cherry and strawberry, which stood out with values of 2000 and 445 µM Trolox.g^−1^, respectively, all fruits had values below 100 µM Trolox/g. The results of a study on the antioxidant capacity of several foods through the ORAC method revealed that the fruits avocado, banana, mango, melon, and guava had values of 19.33, 8.79, 10.02, 3.15, and 19.9 µM Trolox·g^−1^, respectively, which are all below those found for the palm fruits in this study.

Due to its high antioxidant capacity, the methanolic extract from *bacaba* was analysed by LC-DAD-ESI-MS and revealed a major peak with maximum visible intensity at 522 nm at 13.53 min. The peak mass spectrum had a molecular ion [M–H]^+^ *m/z* 595 and fragments *m/z* 449 and 287, which are typical of cyanidin-3-*O*-rutinoside (**1**). After acid hydrolysis of the extract, a single peak with maximum visible intensity at 522 nm and a molecular ion [M–H]^+^ *m/z* 287 and [M–H]^−^ *m/z* 285 was detected and attributed to cyanidin (**10**). The presence of a molecular ion [M–H]^−^ *m/z* 593, was detected. The fruits of two other Amazonian palms, açai (*Euterpe oleracea*) and juçara (*Euterpe edulis*), also have cyanidin-3-*O*-rutinoside as one of their main components [19,32]. The anthocyanins were considered the compounds responsible for the antioxidant action in açai [33], which may also be the case with *bacaba*.

The correlation coefficients among the bioactive compounds and the total antioxidant capacity evaluated through the β-carotene/linoleic acid, DPPH, and ORAC methods are shown in Table 5. Significant positive correlations were found among ORAC and polyphenols (*r* = 0.90), total anthocyanins (*r* = 0.90), and yellow flavonoids (*r* = 0.68). There were significant positive correlations among the β-carotene/linoleic acid method and Vitamin C (*r* = 0.76), polyphenols (*r* = 0.59), total anthocyanins (*r* = 0.54), and yellow flavonoids (*r* = 0.57). As expected, significant negative correlations were found among the DPPH method and the contents of yellow flavonoids and polyphenols. In the bioactive compounds, significant positive correlations were found among the polyphenols, yellow flavonoids, and total anthocyanins.

**Table 5:** Pearson’s correlation coefficients among the bioactive compounds and antioxidant capacity (fresh matter) of edible parts of five Amazon native fruits from palm trees.

R	ORAC	DPPH ^1^	β-Carotene	Vitamin C	Total Polyphenols	Anthocyanins	Carotenoids
Flavonoids	0.68 **	−0.69 *	0.57 *	0.31	0.73 **	0.65 **	0.33
Carotenoids	−0.26	−0.16	0.05	−0.56 *	−0.36	−0.45	
Anthocyanins	0.90 **	−0.54	0.54 *	0.77 **	0.99 **		
TEPP	0.90 **	−0.58 *	0.59 *	0.75 **			
Vitamin C	0.44	−0.20	0.76 **				
β-carotene	0.25	−0.47					
DPPH	−0.59 *						

^1^ No results were found for *pupunha*. ** and * Significative at 1% and 5% probability, respectively, by *t*-test.

Gardener *et al.* [34], while evaluating the contributions of Vitamin C, carotenoids, and total phenolics to the antioxidant capacity of fruit juices, found that the contribution of carotenoids was insignificant and that phenolics seem to be the main compounds contributing to the antioxidant potential of non-citric juices, although its identity and bioavailability requires further investigation. In another study with Citrus fruits, Couto and Canniatti-Brazaca [35] reported that the antioxidant capacity is likely due to the phenolic compounds and Vitamin C. In the present study, we consider that the total polyphenols was the factor that most contributed to the antioxidant capacity of the fruits evaluated.

## 4. Conclusions 

In conclusion, the fruits from the Amazonian palm trees studied stood out as considerable sources of total carotenoids (*tucumã* and *buriti*), total polyphenols (*bacaba* and *tucumã*), and total anthocyanins (*bacaba*), as well as relelvant sources of yellow flavonoids (*bacaba*, *tucumã*, and *buriti*). The fruits that showed a high antioxidant capacity were *bacaba* (through all methods) and *tucumã* (through the β-carotene/linoleic acid and DPPH methods). These results suggest the great potential for the use of native Amazonian fruits as food. 

## References

[1] Horst M.A., Lajolo F.M. Biodisponibilidade de Compostos Bioativos de Alimentos. https://nutrisaude14.files.wordpress.com/2014/09/biodisponibilidade-1.pdf.

[2] Prior R.L., Cao G. (2000). Antioxydant phytochemicals in fruits and vegetables: diet and health implications. Hort. Sci..

[3] Halliwell B. (2006). Reactive species and antioxidants. Redox biology is a fundamental theme of aerobic life. Plant. Physiol..

[4] Volp A.C.P., Renhe I.R.T., Stringueta P.C. (2009). Natural pigments bioactive. Alim. Nutr..

[5] Santos M.F.G., Alves R.E., Roca M. (2015). Carotenoid composition in oils obtained from palm fruits from the Brazilian Amazon. Grasas Aceites.

[6] Santos M.F.G., Alves R.E., Ruíz-Méndez M.V. (2013). Minor components in oils obtained from Amazonian palm fruits. Grasas aceites.

[7] Santos M.F.G., Marmesatb S., Brito E.S., Alves R.E., Dobarganes M.C. (2013). Major components in oils obtained from Amazonian palm fruits. Grasas aceites.

[8] Strohecker R., Henning H.M. (1967). Analisis de Vitaminas: Métodos Comprobados.

[9] Francis F.J., Markakis P. (1982). Analysis of anthocyanins. Anthocyanins as Food Colors.

[10] Higby W.K. (1962). A simplifield method for determination of some the carotenoid distribution in natural and carotene-fortifield orange juice. J. Food Sci..

[11] Larrauri J.A., Rupérez P., Saura-Calixto F. (1997). Effect of drying temperature on the stabilitity of polyphenols and antioxidant activity of red grape pomace peels. J. Agric. Food Chem..

[12] Obanda M., Owuor P.O. (1997). Flavonol composition and caffeine content of green leaf as quality potential indicators of Kenyan black teas. J. Sci. Food Agric..

[13] Marco G.J. (1968). A rapid method for evaluation of antioxidants. J. Am. Oil Chem. Soc..

[14] Brand-Williams W., Cuvelier M.E., Berset C. (1995). Use of free radical method to evaluate antioxidant activity. Food Sci. Technol..

[15] Ou B., Hampsch-Woodill M., Prior R.L. (2001). Development and validation of an improved oxygen radical absorbance capacity assay using fluorescein as the fluorescent probe. J. Agric. Food Chem..

[16] Lin L.-Z., Harnly J. (2007). A standardized method for the identification of glycosylated flavonoids and other phenolic compounds in all plant materials. J. Agric. Food Chem..

[17] Rufino M.S.M., Alves R.E., Brito E.S., Pérez-Jiménez J., Saura-Calixto F., Mancini-Filho J. (2010). Bioactive compounds and antioxidant capacities of 18 non-traditional tropical fruits from Brazil. Food Chem..

[18] Finco F.D.B., Kammerer D.R., Carle R., Tseng W., Boser S., Graeve L. (2012). Antioxidant activity and characterization of phenolic compounds from bacaba (*Oenocarpus bacaba* Mart.) fruit by HPLC-DAD-MS. J. Agric. Food Chem..

[19] Schauss A.G., Wu X., Prior R.L., Ou B., Huang D., Owens J., Agarwal A., Jensen G.S., Hart A.N., Shanbrom E. (2006). Antioxidant capacity and other bioactivities of the freeze-dried Amazonian palm berry, *Euterpe oleracea* Mart. (açai). J. Agric. Food Chem..

[20] Arora A., Muraleedharan G.N., Strasburg G.M. (1998). Structure-activity relationships for antioxidant activities of a series of flavonoids in a liposomal system. Free Radic. Biol. Med..

[21] Loest H.B., Noh S.K., Koo S.I. (2002). Green tea extract inhibits the lymphatic absorption of cholesterol and alpha-tocopherol in ovariectomized rats. J. Nutr..

[22] Souza M.O., Santos R.C., Silva M.E., Pedrosa M.L. (2011). Açaí (Euterpe oleracea Martius): Chemical composition and bioactivity. J. Braz. Soc. Food Nutr..

[23] Mambrin M.C.T., Barrera-Arellano D. (1997). Caracterización de aceites de frutos de palmeras de la región amazonica del Brasil. Grasas Aceites..

[24] Rodriguez-Amaya D.B. (1996). Assessment of the provitamin a contents of foods—The Brazilian Experience. J. Food compos. Anal..

[25] Roesler R., Malta L.G., Carrasco L.C., Holanda R.B., Sousa C.A.S., Pastore G.M. (2007). Antioxidant activity of cerrado fruits. Ciên. Tecnol. Aliment..

[26] Hassimoto N.M.A., Genovese M.I., Lajolo F.M. (2005). Antioxidant activity of dietary fuits, vegetables and commercial frozen pulps. J. Agric. Food Chem..

[27] Duarte-Almeida J.M., Santos R.J., Genovese M.I., Lajolo F.M. (2006). Evaluation of the antioxidant activity using the B-carotene/linoleic acid system and the DPPH scavenging method. Ciên. Tecnol. Aliment..

[28] Koleva I.I., Beek T.A., Linssen A., Evstatieva L.N. (2002). Screening of plant extracts for antioxidant activity: A comparative study on three testing methods. Phytochem. Anal..

[29] Frankel E.N. (1993). In search of batter methods to evaluate natural antioxidants and oxidative stability in food lipids. Food Sci. Technol..

[30] Melo E.A., Maciel M.I.S., Lima V.L.A.G., Santana A.P.M. (2006). Antioxidant capacity of vegetables commonly consumed. Ciên. Tecnol. Aliment..

[31] Wu X., Beecher G.R., Holden J.M., Haytowitz D.B., Gebhardt S.E., Prior R.L. (2004). Lipophilic and hydrophilic antioxidant capacities of common foods in the United States. J. Agric. Food Chem..

[32] de Brito E.S., Araújo M.C.P., Alves R.E., Carkeet C., Clevidence B.A., Novotny J.A. (2007). Anthocyanins Present in Selected Tropical Fruits: Acerola, Jambolão, Jussara, and Guajiru. J. Agric. Food Chem..

[33] Pozo-Insfran D.D., Brenes C.H., Talcott S.T. (2004). Phytochemical composition and pigment stability of acai (*Euterpe oleracea* Mart.). J. Agric. Food Chem..

[34] Gardener P.T., White T.A.C., McPhail D.B., Duthie G.G. (2000). The relative contributions of vitamin C, carotenoids and phenolics to the antioxidant potential of fruit juices. Food Chem..

[35] Couto M.A.L., Canniatti-Brazaca S.G. (2010). Quantification of vitamin C and antioxidant capacity of citrus varieties. Ciên. Tecnol. Aliment..

